# “If everyone comes together, many things can be changed”: A qualitative study on men’s perspectives on menstrual health and equity in the Barcelona area (Spain)

**DOI:** 10.1371/journal.pone.0312685

**Published:** 2025-02-27

**Authors:** Andrea García-Egea, Constanza Jacques-Aviñó, Anna Berenguera, Jordi Baroja-Benlliure, Diana Pinzón-Sanabria, Anna Sofie Holst, Tomàs López-Jiménez, Jordina Munrós-Feliu, María Mercedes Vicente-Hernández, Carme Valls-Llobet, Cristina Martínez-Bueno, Laura Medina-Perucha

**Affiliations:** 1 Fundació Institut Universitari per a la recerca a l’Atenció Primària de Salut Jordi Gol i Gurina (IDIAPJGol), Barcelona, Spain; 2 Universitat Autònoma de Barcelona, Bellaterra (Cerdanyola del Vall̈s), Spain; 3 Network for Research on Chronicity, Primary Care and Health Promotion (RICAPPS), Spain; 4 Departament d’Infermeria, Universitat de Girona, Girona, Spain; 5 Centre Jove d’Atenció a les Sexualitats, Barcelona, Spain; 6 SomiArte Taller, Barcelona, Spain; 7 Atenció a la Salut Sexual i Reproductiva (ASSIR) Muntanya/La Mina, Institut Català de la Salut, Barcelona, Spain; 8 Sexual and Reproductive Health Care Research Group (GRASSIR), Barcelona, Spain; 9 Centro de Análisis y Programas Sanitarios (CAPS), Barcelona, Spain; 10 Servei d’Atenció a la Salut Sexual i Reproductiva (ASSIR), Direcció Assistencial d’Atenció Primària, Institut Català de la Salut, Barcelona, Spain; 11 Universitat de Barcelona, Barcelona, Spain; Rural Women’s Social Education Centre, INDIA

## Abstract

There is a paucity of research exploring how men and individuals who do not menstruate comprehend menstrual health and equity. The objective of this study was to explore the conceptualization and attitudes towards menstruation and the menstrual cycle among men and people who do not menstruate aged between 18 and 55 in the Barcelona area. Furthermore, it examined their involvement in menstrual health and equity initiatives. This qualitative study employed a feminist critical perspective. Seventeen semi-structured photo-elicitation interviews were conducted. It was frequently observed that menstrual education was often inadequate and biomedical in nature. Participants often acquired knowledge about menstruation through interactions with menstruating sex-affective partners. Menstruation was generally perceived negatively, and menstrual taboo and stigma were apparent. Some participants expressed the view that men should raise awareness of a more positive stance on menstruation. The findings of this study highlight the need for structural menstrual policies and community programs where men and people who do not menstruate are involved.

## Introduction

The menstrual cycle and menstruation are not only biological processes but also sociocultural phenomena [1, 2]. The existence of menstrual taboos, stigma and discrimination has resulted in these processes being relegated to the private sphere, particularly away from the gaze of men [3]. Androcentric approaches to health, politics, society and research have played a significant role in rendering menstruation and the menstrual cycle invisible.

The recent increase in public and academic attention towards menstruation and the menstrual cycle in the last few years has led to the consideration of these processes as vital signs of health [4]. A recent definition of menstrual health has included a less clinical and a more holistic view (based on the guarantee of access to menstrual information and products, to appropriate healthcare services as well as experiences free of stigma and psychological discomfort) [5].

There is a notable dearth of research on menstrual knowledge and attitudes among men and non-menstruating individuals [6]. People who do not menstruate (PNM) are defined as individuals who do not experience a menstrual cycle or menstruate and do not identify their gender as “male”. This includes, but it is not limited to, trans women, non-binary individuals, or intersexual people who do not menstruate. Nevertheless, the existing evidence has underscored the necessity to incorporate the perspectives of men and PNM in menstrual-related initiatives [7–9]. Some studies indicate that men may obtain menstrual information from school-based learning programs or through conversations with women [7, 8]. The available evidence indicates that the lack of knowledge among boys contributes to the perpetuation of stereotyped conceptions surrounding menstruation [10]. Studies conducted in the United States [7] have shown that boys typically learn about menstruation through their sisters, mothers, friends, or menstruating partners. Additionally, studies conducted in the United States and Australia have demonstrated that adult men tend to develop a more positive perception of menstruation when they have sex-affective relationships with menstruating partners [11, 12].

The implementation of menstrual education based on a biomedical perspective, which prioritizes the anatomy, physiological and reproductive processes associated with menstruation and the menstrual cycle, did not result in a reduction in the occurrence of menstrual teasing and stigmatization among young people in Tanzania [13]. The biomedical approach to menstrual education may inadvertently reinforce cultural and social norms related to menstruation [14]. This could potentially perpetuate menstrual myths and misconceptions. The perpetuation of patriarchal structures is reinforced by menstrual beliefs, which privilege boys and men, and utilize this differentiation as a mechanism of dominance over women [11]. The majority of these beliefs identify women as being "excessively emotional", particularly in relation to “negative” emotions (e.g. anger) during menstruation [15]. Some myths portray women and people who menstruate (PWM) as symbols of impurity, due to the cultural and historical associations with menstrual blood as a source of impurity. In some countries in the Global South, menstruating women are excluded from the use of menstrual management facilities (e.g. bathrooms) and are also excluded from participation in several spheres of life [16]. Investigations conducted in both from the Global South and North have revealed that young girls and women are subjected to ridicule by others during menstruation [9, 17]. This phenomenon has the potential to exacerbate existing gender-based health inequities, which may have ramifications for the well-being of women and PWM.

Negative attitudes towards menstruation among men can perpetuate stigma and myths surrounding menstruation [18], which underscores the importance of investigating their knowledge and attitudes towards menstruation, women and PWM. This research has the potential to be pivotal in promoting the involvement of men and PNM in menstrual actions, from research to policy development. The present study aimed to explore the conceptualization and attitudes towards menstruation and the menstrual cycle among men and PNM between the ages of 18 and 55 in the Barcelona area, as well as their role in menstrual health and equity actions.

## Materials and methods

This is exploratory qualitative study employs a critical feminist perspective, with a focus on examining the influence of androcentric and patriarchal systems can have an impact on menstrual health and equity. This approach is conducive to the undertaking of critical analyses, which entail the questioning of dominant structures [19] and the exploration of the ways in which the organization of power and institutions can give rise to the exclusion and oppression of certain individuals and social groups. The feminist perspective is concerned with addressing gender inequities, with the aim of eliminating the inherent discrimination against women and other oppressed groups and promoting social equity and justice [20]. In order to avoid the exclusion of non-menstruating individuals who do not identify as men (e.g. trans, intersexual or non-binary people), the present study will use the terms “men” and “people who do not menstruate” (PNM). Similarly, the terms "women" and "people who menstruate" (PWM) will be used to ensure the inclusion of people who are menstruating but do not identify as a “woman”.

With regard to the research team, 10 authors identify their gender as “women” and 2 authors as “men”. The interviews were conducted by women, and the majority of the data analysis was conducted by women members of the team. The entire research team (men and women) participated in the conceptualization of the study, the development of the materials, and the analysis and interpretation of the data (particularly in defining the final analytical framework).

### Participants

The study participants were seventeen men, aged between 19 and 54 years old, residing in Barcelona or the surrounding areas. The participants were selected between the ages of 18 and 55 in order to gain a general overview of menstrual perceptions within the population under study. Furthermore, this age group was selected to maintain consistency with a previous qualitative exploratory study conducted with women and PWM, as part of the same project.

The majority of participants had completed university studies and were employed in full- or part-time positions. Four participants reported being born outside of Spain. With regard to sexual orientation, thirteen participants were identified as heterosexual (see Table 1). Individuals who were biologically capable of menstruating throughout their life cycle were excluded from this study. Despite the study’s non-binary approach to gender, the term "men" was used to refer to participants, as they all identified as men.

**Table 1:** Participants’ sociodemographic characteristics (N = 17).

ID	Age	Residence (city)	Birthplace	Administrative status	Employment status	Economic issues in the last year	Completed education	Gender	Sexual orientation	Interview location^a^	Identified as trans
P1	27	Barcelona	Spain	Spanish nationality	Works part-time	Yes, sometimes	Secondary education	Man	Heterosexual	[redacted for blind review]	No
P2	19	Barcelona	Spain	Spanish nationality	Studies full-time	No	Secondary education	Man	Heterosexual	[redacted for blind review]	No
P3	19	Cornellà de Llobregat	Spain	Spanish nationality	Unemployed	No	Professional education	Man	Heterosexual	[redacted for blind review]	No
P4	24	Esplugues de Llobregat	China	Spanish nationality	Works full-time	Yes, sometimes	Professional education	Man	Heterosexual	[redacted for blind review]	No
P5	37	Tordera	Macedonia	Permanent residence	Works full-time	No	University studies	Man	Homosexual	[redacted for blind review]	No
P6	49	Barcelona	Belgium	Spanish nationality	Works full-time	No	University studies	Man	Heterosexual	[redacted for blind review]	No
P7	48	Sabadell	Spain	Spanish nationality	Works full-time	No	University studies	Man	Heterosexual	[redacted for blind review]	No
P8	23	Barcelona	Spain	Spanish nationality	Works full-time	Yes, sometimes	University studies	Man	Homosexual	[redacted for blind review]	No
P9	46	Badia del Vallès	Spain	Spanish nationality	Works full-time	No	Elementary education	Man	Heterosexual	[redacted for blind review]	No
P10	27	Barcelona	Lebanon	Temporal residence	Works full-time	Yes, sometimes	University studies	Man	Heterosexual	[redacted for blind review]	No
P11	54	Sant Adrià del Besòs	Spain	Spanish nationality	Works full-time	No	University studies	Man	Homosexual	[redacted for blind review]	No
P12	23	Barcelona	Spain	Spanish nationality	Works part-time	Yes, sometimes	University studies	Man	Heterosexual	[redacted for blind review]	No
P13	25	Barcelona	Spain	Spanish nationality	Works full-time	No	University studies	Man	Heterosexual	[redacted for blind review]	No
P14	35	Barcelona	Spain	Spanish nationality	Works part-time	No	University studies	Man	Heterosexual	[redacted for blind review]	No
P15	26	La Palma de Cervelló	Spain	Spanish nationality	Works part-time	No	University studies	Man	Heterosexual	[redacted for blind review]	No
P16	28	Barcelona	Italy	Permanent residence	Works part-time	Yes, sometimes	Elementary education	Man	Bisexual	[redacted for blind review]	No
P17	31	Sabadell	Spain	Spanish nationality	Works full-time	No	University studies	Man	Heterosexual	[redacted for blind review]	No

^a^[redacted for blind review]

### Sampling and recruitment

The sampling was purposive, although the researchers were mindful to ensure discursive diversity by recruiting participants of different age groups, sexual orientations and birthplaces. The younger participants were recruited through the [name of recruitment center], a sexual youth health center in Barcelona, while the older participants were recruited through key contacts related to the health sector. Additionally, snowball sampling techniques were employed. Due to the time constraints and the lack of identification of institutionalized spaces to locate potential participants, the study employed a pragmatic sampling approach.

### Data collection

In total, 17 semi-structured photo-elicitation interviews were conducted between December 2021 and February 2022. The use of photo-elicitation proved an effective method for enhancing reflexivity processes and facilitating emotionally and challenging conversations and taboo topics, including those related to menstruation and associated stigma. This technique facilitates the collection of more detailed data and serves to reinforce the themes identified in regular interviews [21]. A topic guide was developed (see S1 File) and two photographs were selected for use in the interviews. The materials were prepared by the research team in accordance with the study’s objectives. Prior to the commencement of fieldwork, the topic guide was trialed with two male participants. The first photograph portrays a group of women engaged in a marathon event, with one individual exhibiting visible menstrual bleeding through her clothing. The second photograph depicts Mexican congresswomen and representatives from another Mexican organization advocating for menstrual rights. They are holding signs with messages such as “Menstruation is not a luxury”. The images were readily comprehensible and interpretable by the study participants.

The interviews were conducted in person (N = 14) and via telephone (N = 3). In the case of telephone interviews, the photographs were sent via telephone at the outset of the interview. A total of 16 interviews were conducted by the first author, while one additional interview was conducted jointly by the first and last author. Prior to the commencement of the interviews, all participants provided verbal and written informed consent. Additionally, participants completed a questionnaire to indicate their sociodemographic data (see Table 1). The interviews were conducted for a duration of between 35 and 75 minutes and were audio-recorded for subsequent transcription. As a gesture of appreciation, each participant was presented with a 10-ride metro card, valued at 10 euros.

### Data analysis

The data were subjected to analysis using the Thematic Analysis approach outlined by Braun and Clarke [22]. Firstly, the first author undertook the transcription of all interviews in their original form. Secondly, the first author undertook the coding of 12 interviews, with a further three interviews being subjected to a process of triangulation by the second and last authors. Subsequently, an initial set of themes and subthemes were inductively identified by the first and last author through a process of collaborative analysis, critical reflection, and discussion. A meeting was held with the remainder of the research team to discuss the preliminary conceptual framework, including the identified themes and subthemes. The remaining five interviews were then subjected to coding by the first author and triangulated with two of them by the last author. The analysis was based on the conceptual framework developed previously, considering the discussions held with the research team. No new themes or subthemes were identified in this phase of analysis, but new information emerged that was incorporated into the initial framework. Subsequently, a second discussion with the research team was conducted to establish a final thematic framework.

To ensure the quality and rigor of the research, the Guba and Lincoln criteria [23] and the Critical Appraisal Skills Programme tool [24] were employed (S2 File).

### Ethics statement

The study obtained the requisite ethical approval from the IDIAPJGol Ethical Committee on 20 November 2019 (Ref 19/178-P). Prior to the commencement of the interviews, all participants were duly informed of their right to consent to participate in the study, either verbally or in writing. All participants were informed of the conditions of anonymity, confidentiality, and the right to withhold information, as well as the option to withdraw from the study or delete their data prior to analysis.

## Results

### “"We talked […] two or three lines about menstruation. […]. The biological part": Between disinformation and reductionist approaches to menstrual education

#### “No one explained it to me, I found out on my own”: Menstrual education as accidental

The participants indicated that their initial experiences with menstruation occurred when their classmates menstruated at school or when they inadvertently observed menstrual products (e.g., in a bathroom cabinet): *"My mother had her pads there* [in the bathroom], *tampax*, *and I slowly*, *later on I learnt*, *I made the association* [with menstruation], *because one day I said ‘damn*, *this is for my mother for her period to put it here*, *not to stain or anything like that’*, *but no one explained it to me*, *I found out on my own*, *you know*?*”—P15*. The majority of participants acquired their initial knowledge about menstruation during adolescence, between the ages of 11 and 14. This was frequently subsequent to the onset of menstruation among women and other women of a similar age.

The participants perceived the menstrual education they received, particularly within the school environment, to be inadequate. This has led to a lack of accurate information about menstruation and the menstrual cycle more generally. Menstruation was frequently addressed within the context of pregnancy prevention and sexual health programs. As they grew older, participants began to contextualize the acquisition of information through socializing with women and PWM, sometimes through friends but primarily through their sex-affective partners who menstruate: *"Well*, *at the beginning* [menstruation was discussed] *by friends who introduced me to the subject*, *but when I came to talk about it more was with my ex-partner"—P1*. Menstrual conversations with women and PWM during adulthood frequently occurred in informal, day-to-day situations. This was often in response to perceived barriers to sexual activity or social participation caused by menstruation: *"It comes up in the conversation because sometimes there are logistical needs*, *let’s say*, *but it’s not something you say*: *‘Let’s talk about it* [menstruation]*’*. *No*. *This has never happened in my case"—P11*.

The majority of participants had no prior experience of discussing menstruation or had only done so in a cursory manner within the family context. A small number of participants indicated that they had derived information from television, given their awareness that advertisements were responsible for perpetuating a stereotypical approach to menstruation: *“You listened to the ad*, *you saw the people that was content and happy with the pads*, *well those ads done back then*, *and they didn’t go beyond the problem*. *Well*, *the problem or difficulty or what it* [menstruating] *meant”- P6*. Some participants concurred that social media has the potential to disseminate messages that challenge the stigma associated with mental health issues.

#### “Now I see a more social dimension to it”: Biomedical and sociostructural perspectives on menstruation

The initial menstrual information received by participants was predominantly biomedical in nature. This was particularly evident in formal settings, where the content was largely focused on menstruation in the context of reproduction and sexual encounters: *"I remember at some point in high school discussing sexuality*, *we talked about masculine and feminine genitalia*, *a bit*, *two or three lines about menstruation*. *It [menstrual bleeding] lasts these many days*, *and so on*. *The biological part*.*"- P8*.

The majority of participants indicated that their understanding of menstruation had evolved from an initial biological perspective to a more sociostructural one, encompassing the emotional and social implications of menstruating. They attributed this shift in perspective to their interactions with other women and individuals who identify as PWM: *"What gives me more knowledge is to continue sharing spaces with women and also growing in terms of age*, *because I also understand that perceptions change and this will give me more information*.*”—P12*. Nevertheless, the discourses of the participants remained distinctly biomedical in their perspective on menstruation, regarding it as an exclusively biological phenomenon. Furthermore, a more pronounced biological perspective was evident among those who lacked sex-affective relationships with women and PWM. This lends support to the notion that social learning for men occurs within the context of sex-affective relationships with women and PWM.

### “Menstruation is not a common thing”: Menstrual conceptualizations

#### “She will be back being herself in a few days”: Menstruation as a stigmatized burden

Additionally, a persistent duality was evident in the participants’ narratives. Concurrently with their expression of negative views on menstruation and its implications, they also presented alternative discourses to mitigate the stigmatizing (and occasionally discriminatory) attitudes they espoused. Menstruation was predominantly perceived in a negative light, although on certain occasions, participants regarded it as a natural process. They characterized menstruation as "unpleasant" or "disappointing", associating it with pain: *“Everything else* [what it means to menstruate] is *fucking shit*, *you know*? *You say to me*, *‘Do you wish you were a woman to be able to have this* [menstruation]?*’ And I say*, *’No*, *because I would have had my period for 40 years’*. *I wouldn’t like to have the period every month*, *you know*, *it’s a problem that I wouldn’t like to manage*.*"—P14*.

Menstruation is associated with a series of physical and emotional fluctuations that were predominantly perceived by participants as negative changes. These changes included feelings of tiredness, fatigue, aggression, irritability, nervousness, irascibility and a greater emotional lability. Some participants also revealed how emotionality among women and PWM may be solely associated with the menstrual cycle. This can contribute to invalidating their emotional experiences and may even result in infantilization, depersonalization and stigmatization of women and PWM: *“Poor girl*, *she has that complicated week and she is also in a bad mood*. *Listen*, *poor girl*, *because there is a hormonal turmoil*. *It’s okay*, *she will be back being herself in a few days" -P7*.

A few participants perceived menstruation as a potential obstacle to sexual encounters. As P7 observed, some men appeared to view menstruation as a hindrance to accessing women and PWM bodies, and as a means of satisfying their own sexual desires or impulses: *"If you are with a stable partner*, *this obviously*, *there are times when you regret having to deprive yourself of certain sexual needs*, *desires or sexual impulses*, *but you perfectly understand that this it’s not the time" -P7*. Those who did not have sex-affective relationships with women and PWM indicated a certain distance with menstruation, thereby supporting the notion that men’s experiences of menstruation are particularly influenced by their sexual partners.

The reproductive function of menstruation was the most frequently referenced positive aspect of this process: *"Well*, *the ultimate purpose of menstruation is to create life*, *you know*? *It can be positive*, *if it’s within your goals or your purpose*, *let’s say*. *It’s a capacity that women have that men don’t have*. *And it’s a very beautiful capacity [*…*] the bond I have with my mother*, *I don’t know if it’s because she gave birth to me*, *but it hasn’t been the bond I have never had [(…] with my father*.*”—P14*. A number of participants linked menstruation with the capacity to bear children. This may suggest that the processes of gestation and reproduction are viewed as being the domain of women, with men perceived to have a less active or responsible role in these matters: *"Well*, *because I don’t menstruate*, *so I can’t have a child*. *Someone who menstruates can do it*, *so*, *then this* [menstruation] *is a very positive thing*.*"—P3*.

A number of participants asserted that menstruation can serve as an indicator of overall health and well-being. Another participant posited that menstruation can facilitate body literacy for women and PWM: *"My partner*, *[*…*]*, *it often puts her at ease to say*, *‘In the last few days*, *I’ve been very hungry*, *I’ve been very anxious and that’s because my period has to come or my period has come‴—P12*.

#### “Your coquettish femininity is quickly dismantled by menstruation”: Womanhood, femininity and menstruation

In general, participants associated menstruation with a distinctive experience of the "female gender," conceptualizing gender in a binary manner. This seemed to reinforce their perception of menstruation as an identity attribute of women, depicted as a monolithic entity. Notably, P3 observed that menstruation was not a prevalent experience, suggesting that not menstruating (e.g., the experience of men and PNM) might be the socially normative one: *"Just because of the fact that it* [menstruation] *is not a common thing" -P3*. Furthermore, P7 emphasized the incongruence between menstruation and conventional notions of femininity, particularly in relation to the attributes of politeness and cleanliness, which were perceived as hegemonic feminine characteristics: *“You go dressed up and it* [menstruation] *catches you off guard and your glamour suddenly disappears*, *doesn’t it*? *Your coquettish femininity is quickly dismantled by this dirty trick*, *this of menstruation*.*"—P7*.

All participants indicated that menstruation was not a topic of discussion in a male environment. When men did engage in discussions about menstruation, they often did so in a stigmatizing manner, associating it with emotional fluctuations and linking it to sexual encounters: *"It’s a topic that I have hardly ever heard men talk about or talk about it knowledgeably [*…*] The typical one*: *‘Oh be careful*, *she’s on her period’*. *Nonsense like that*. *‘Look at that bad-tempered face’ [*…*] Or talk about sexual topics and I don’t know*, *like ‘I don’t like it [having sex during menstruation]’*.*”—P1*

#### “It disgusts them”: Menstrual taboo, stigma and discrimination

The participants concurred that menstrual taboo constituted a social reality within their context, although they perceived a recent diminution in its prevalence. P13 conceptualized menstrual taboo as an "intersectional" phenomenon, encompassing disparate forms of taboo (e.g., those pertaining to blood or sexuality) and gender inequities: *"It condenses blood*, *which is also a taboo*, *the woman’s body*, *sexuality and everything*. *In the end*, *it is something that encompasses many things so that it turns out to be a taboo" -P13*. Furthermore, other participants identified additional factors that contribute to the formation of menstrual taboos, including cultural, geographical, religious, political, and social network contexts: “*Even though my parents are engineers*, *so they’re really educated and everything*, *but of course it’s the country*, *the culture here*, *that you don’t talk about these things*.*”—P5*.

In contrast, participants explicitly stated that menstruation and the menstrual cycle were not considered taboo subjects. However, the use of euphemisms such as "there" or "I have to go down here" to refer to menstruation or the genitals was stated in their narratives, indicating that some men may be unaware of the taboo nature of these topics. Furthermore, participants asserted that they do not belong to the group of men who perpetuate menstrual taboo and stigma. For instance, participants spoke in the third person, referring to "men" or "my friends," which may have been a way of eluding themselves: *"Friends and acquaintances who are directly disgusted by it* [menstruation].*"—P13*.

In the context of the photo-elicitation, the initial photograph prompted a discussion among the male participants, who generally perceived menstruation (and particularly staining) in public as "an act of courage". However, this perception was accompanied by a simultaneous recognition of the discomfort and shame associated with the experience. Additionally, some participants made comments that diminished the woman in the photograph, portraying her as a victimized and childlike figure: *“Well*, *it [menstruating in public] sucks*. *Poor girl*. *If she wanted to do it [menstruating in public] because she hasn’t put anything on [menstrual products]*, *I would say ‘well done’*. *If she was not conscious*, *it’s bullshit*. *What a shame*.*”—P3*.

The participants did not report witnessing discriminatory practices against women and PWM on the basis of their menstruation. However, as previously noted, they did report hearing disparaging remarks from other men. A few participants expressed the view that sexism is a phenomenon that has receded into the past, citing the current favorable sociocultural context for women: *"In the past*, *there was a masculinity that would make you freak out*, *women were at home*. *Now we are ‘hyperfeminists’”—P3*. In turn, other participants reflected on the role of hegemonic internalized sexisms as a vestige of patriarchal societies: *“In the end there are things from their previous societies that have still been perpetuated* […] *and we still need to work deeply to change these dynamics*.*" -P15*

### “The state should make moves and do its best”: Menstrual policies and social changes

#### “When we talk about money, we don’t focus on menstruation”: Resource prioritization and menstrual policies

In general, participants demonstrated a lack of awareness regarding current menstruation policies. None of the participants were able to provide an accurate definition of the term "menstrual poverty". As P3 explained, while he may engage in discussions about financial matters with his friends, the topic of menstrual finances is never broached.

It was observed by several participants that the absence of menstrual policies is linked to androcentric societies, in which women, PWM and menstruation have been historically regarded as non-normative and marginalized: *[Menstruation] is not considered a basic issue because of the patriarchy*, *because it is considered ‘a women’s thing’*. *Sometimes women are almost included in the minority category [*…*] And it’s like*, *’if they are the 50% of the population*, *you can’t put them in the minority category*.*’”—P13*.

The majority of participants concurred with the proposal to prioritize the allocation of public resources towards the enhancement of menstrual education within the school curriculum. It was posited that menstrual education should be made available from an early age, with a particular emphasis on the psychosocial and structural aspects of menstruation, and that it should be accessible to students of both genders: *"Explain it to boys and girls from a very young age*, *and even more to boys because they won’t experience it*. *I don’t know*, *maybe disseminating not so much about periods*, […] *but also how normal girls experience periods and what relationship they have with it and what it implies to them*.*"—P12*. In contrast, two participants (P3 and P17) posited that at some point, menstrual education for men would have to be different.

In discussing the second photo-elicitation image, participants concurred that menstrual products are a necessity, and that reducing taxes on such products could be an avenue for promoting social justice: “*I think that in the health area*, *this (menstrual products) should be free*, *of course it depends on many things*. *But I think that health things should be free and open to the people*.*”- P4*. The provision of subsidized menstrual products for specific demographic groups, including women and PWM in situations of socio-economic vulnerability, emerged as a potential solution to address menstrual inequity in other interviews: *"There are many social groups that are not in a financial situation to be able to buy everything they need to take care of themselves and I think it’s something that the State should make moves and do its best*.*"- P2*.

The topic of menstrual policies in the workplace gave rise to a divergence of opinions among participants. Some participants expressed support for the promotion of such policies, whereas the majority exhibited a degree of reservation. They argued that the endorsement of workplace policies (such as those pertaining to menstruation) should be contingent upon an evaluation of their impact on business productivity and financial stability. Conversely, other participants posited that menstrual policies in the workplace could potentially serve to reinforce existing gender inequities: *"I can see more benefits of handling the taxation of these* [menstrual] *products*, *of offering them at a better price*, *instead of* [menstrual] *leaves*, *because in the end the economic impact would be very high*. *This would somewhat dismantle the claim for gender equality in workplaces*.*"—P7*.

#### “If everyone comes together, many things can be changed”: The role of men in reaching menstrual equity

Some men posit that the relationship between men and women and those who identify as PWM must be founded upon empathy. This approach allows men to identify and attend to the needs of women and those who identify as PWM: *"I believe that the best role is to become aware and empathize [*…*] because in the end these are social changes and for the changes to occur*, *awareness has to generally raise*.*"—P14*. Nevertheless, despite their stated commitment to empathy, they frequently exhibited paternalistic attitudes and framed empathy as an instinctive response to socially acceptable discourse: *"In the end you say*, *if you could do something about it*…*but you can’t*. *You say ’calm down*, *relax’*. *It is what it is*. *‘What do you want to do about it*?*’” -P9*. Nevertheless, a small number of participants exhibited a somewhat divergent attitude. One individual, for instance, articulated that his lack of menstruation rendered him "ignorant" and "useless," explicitly and humbly alluding to his lack of knowledge regarding the experience of menstruation: *"When I see a girl who is suffering*, *who is having a hard time*, *I’m completely ignorant because I don’t know what’s going on and I can’t relate to it*. *I can’t know how it feels*. *I don’t know*, *I try to help but I feel useless at the same time*. *[*…*] I want to know how it feels*, *you know*?*”- P10*.

The participants expressed the view that the involvement of men and women in social and political developments related to menstruation was a crucial factor: *"If everyone comes together*, *there is a majority*, *many things can be changed*.*"- P4*. Additionally, a number of participants asserted that men should not be the primary advocates for menstrual equity, suggesting that their role should be supportive of women. However, a couple of participants (P3 and P17) espoused the view that power should be shared between men and women in menstrual actions. In particular, P3 expressed a certain degree of discomfort if women led social and political actions towards menstrual equity. He also expressed disagreement with the notion that men should not be in a position of power, viewing this as an act of discrimination: *"If they don’t give a role to a person who does not menstruate*, *you are discriminating against them [*…*]*. *I don’t agree*, *I only see girls [referring to the content of the second photo-elicitation image]"-P3*.

Conversely, some participants asserted that there is a necessity for community-based and day-to-day actions: “*Instead of saying*, *’I’m complaining because this person is menstruating*,*’ saying*, *’What can I do to make it easier for this person*?*’”–P16*. P13 posited that confronting stigmatizing and discriminatory attitudes among men could be a pivotal step in addressing menstrual stigma and discrimination. P10, in turn, proposed that the responsibility for driving change among men resided with those in positions of greatest influence, suggesting a lack of ownership in their role in addressing menstrual inequity.

## Discussion

There is a substantial body of research on the sociocultural taboo of menstruation [6–9], which is also evident in our findings. Despite the explicit assertion by participants that menstruation was not a taboo subject, the use of euphemistic language was frequently observed in their references to menstruation. This pattern, observed in a recent study with women and PWM in our context, suggests that menstrual taboo is structurally rooted and normalized in our society. The menstrual taboos identified in our research have been theorized to be designed to preserve hegemonic androcentric socio-political systems [25], as well as to invisibilize women and PWM and the menstrual cycle [17].

The processes of menstruation and the menstrual cycle are rendered invisible within the structures of patriarchy and androcentrism, which effectively prevent them from being considered normative [26, 27]. The conceptualization of menstruation among the male participants in our study was frequently influenced by the perception of menstruation as non-normative. This notion is reinforced by the assumption that a "normative" experience is an inherent aspect of male identity. As Gloria Steinem observed in 1978, the social organization of society would be structured around menstruation if this were a common experience among men [28]. It is noteworthy that women and PWM frequently adopt a similar approach to conceptualizing menstruation and menstrual experiences, indicating the institutionalization of menstrual symbolism and social imagery [11, 17, 29, 30]. It can therefore be seen that, as the participants themselves highlighted in their narratives, menstruation is stereotypically thought to be an identitarian trait of ’what constitutes to be a woman’. This maintains a binary perspective of gender and constrains women and PWM within a hegemonic, limiting and sexist experience. The categorization of menstruation as a "female" experience results in the exclusion of those who do not identify within a gender-confirming binary. Consequently, the health and wellbeing of trans, non-binary and intersexual individuals who menstruate may be overlooked, including within healthcare environments, which could have an adverse impact on their health [31].

The invisibility of menstruation and the menstrual cycle serves to reinforce the negative imaginary that surrounds these processes [2]. The notion that menstruation is predominantly negative has been previously discussed in the menstrual literature, as evidenced by the works of men and women such as [7, 17, 27]. This negative imaginary serves to reinforce the stigmatized vision of women as "monstrous" (inhuman, animal-like and out of control) [32]. Consequently, menstruation is deemed incongruous with the prevailing social expectations, norms, and idealized notions of femininity. Although the majority of participants expressed negative views about menstruation, they also engaged in discourse that was more socially acceptable. This resulted in a disjointed discourse that was potentially influenced by the fact that the interviewer was a woman discussing menstruation and gender-related topics. Men in our study may have been confronted with their own attitudes, aware of the current societal changes towards a more equitable society in which men’s privileges and hegemonic expressions of masculinity are being profoundly questioned.

Reproduction was historically perceived as the primary beneficial aspect of menstruation. In some instances, this appeared to indicate a certain neglect of men’s reproductive and caring responsibilities. This detachment with regard to caregiving has been the subject of extensive study from a gender perspective. Connell posits that hegemonic masculinity is a gender expression that encompasses a set of defining practices, often rooted in domination [33]. These include independence, competitiveness, and the integration of an instrumental and non-affective role in relationships with others. These hegemonic constructions of masculinity, when viewed through a gender binary lens, result in men being positioned as secondary in biological reproduction and caregiving [34]. Consequently, these functions are primarily attributed to women [35].

The discourse surrounding menstruation is characterized by a paternalistic, victimizing and infantilizing approach (e.g. “Poor girl, she has that complicated week and she is also in a bad mood.”-P7). Furthermore, the subjective experiences of women and PWM were invalidated by some participants. This is grounded in the patriarchal model that perpetuates the objectification of menstruating bodies, the devaluation of women’s and PWM’s emotions and experiences, and the perception of women (and PWM) as being physically, morally and emotionally inferior to men [36–38]. Nevertheless, previous research indicates that some men in this study expressed a desire to gain further insight into menstrual experiences [8, 12]. It is proposed that these "alternative" discourses are aligned with non-hegemonic masculinities, which are characterized by a questioning of emotional and social responsibilities [39]. This includes social reproduction, which encompasses self-care and care for others (childcare and elder care) while maintaining physical spaces and organizing required resources (cleaning, shopping, repairing), and human reproduction (bearing and rising children) [40, 41]. However, it is crucial to acknowledge the possibility that some participants may have merely reproduced these discourses to appear "alternative" and align with the perceived social expectations of the interview. As evidenced by this research, challenging dominant discourses and promoting co-responsibility in social reproductive roles could significantly contribute to gender equity.

Menstrual education is frequently constrained to the realms of sexual and reproductive health, with an emphasis on a biomedical perspective. Furthermore, it is typically confined to the private domain and seldom addressed in formal educational settings. Similarly, the findings of this study align with those of previous research, indicating that men primarily acquire knowledge about menstruation from sex-affective partners who menstruate, particularly within the context of sex [7]. In contrast, participants who had not engaged in sex-affective relationships with women or PWM demonstrated a notable disengagement towards menstruation and a more pronounced lack of information. It is therefore recommended that menstrual education be made available in formal settings, with a view to ensuring equitable access to information and resources on this topic. Concurrently, community-based research and initiatives are essential to reinforce the provision of high-quality menstrual education and guarantee menstrual equity. Indeed, the majority of participants considered the creation of comprehensive menstrual education policies integrated into the school curriculum to be a pivotal initial step in improving access to menstrual information. Furthermore, it is essential to challenge the prevailing menstrual conceptions and negative attitudes. It is imperative that these policies include men, given their pivotal role in perpetuating and disseminating menstrual stereotypes [11, 18]. The prevailing biomedical and reproduction-based approach to menstruation should be superseded by menstrual education that addresses menstruation as a social, cultural and political issue [2]. This should entail the promotion of body literacy, menstrual management [18] and self-care, as well as the tackling of menstrual taboos, stigma and discrimination. Menstrual education should extend beyond the gender binary to guarantee the rights of PWM.

The views on workplace menstrual policies were found to be conflicting by some participants. These findings suggest the existence of barriers preventing some men from deconstructing their social privileges, questioning their status quo, and contributing to a more equitable society in which women and PWM have the necessary resources and spaces to manage menstruation. Therefore, it can be concluded that workplace menstrual policies will only advance gender equity if they are adopted in spaces committed to challenging menstrual stigma and cultural beliefs [42, 43].

### Strengths and limitations

This study’s primary strength is that it offers, to the best of our knowledge, the first qualitative data on menstrual equity and health, including the perspectives of men in our context. The findings of this study have the potential to influence the awareness of researchers and policymakers regarding the significance of incorporating men as active advocates for menstrual policies and community-based initiatives. Another strength of this study is the use of photo-elicitation techniques, which enabled us to gain deeper insights from the male participants. Furthermore, these techniques facilitated the exploration of sensitive and often taboo topics, such as menstruation and the menstrual cycle. Additionally, this study offers an in-depth examination of men’s perceptions and attitudes from a feminist perspective.

It is also important to consider the limitations of this study. The initial challenge was participant recruitment, as the research topic did not elicit sufficient motivation to encourage involvement. As is the case with any other research, the participants demonstrated a personal interest in the research topic. Consequently, the findings may not be illustrative of men who might be less interested or who hold more stigmatizing and sexist views on menstruation, women and PWM. Furthermore, the inclusion of specific profiles (e.g. non-binary or trans individuals, or men/PNM in a situation of socioeconomic vulnerability) was hindered due to the aforementioned recruitment challenges. Additionally, both interviewers were women, which may have influenced the responses of the male participants, prompting them to provide answers that were more socially desirable.

## Conclusions

To the best of our knowledge, this is the first study to explore men’s sociocultural meanings and attitudes towards menstruation in the Spanish context. The research findings indicate that men tend to hold negative and stigmatizing views about menstruation. The implementation of policies that challenge the stigma surrounding menstruation and promote gender equity has the potential to foster an equitable society. The efforts made in recent years to promote gender-based community action and research have the potential to be the driving forces behind the commencement of a paradigm shift that is more menstrual-friendly. Nevertheless, there is a long journey ahead. It is imperative to elucidate the way men and PNM can provide assistance to women and PWM in spearheading menstrual initiatives. The formulation and implementation of structural and multicomponent menstrual strategies in educational, health, workplaces and other settings are crucial for the promotion of menstrual health and the reduction of menstrual inequities. It is imperative that policymaking is evidence-based and that isolated and superficial menstrual strategies are avoided. Such strategies often become *tokenistic* and can have unintended negative consequences. It is recommended that menstrual education be provided in both school and community settings. It is also imperative that men and PNM be included in menstrual strategies, in order to enhance the efficacy and extend the scope of menstrual policies. It is imperative to consider and address the opposition of some men, particularly in the context of workplace menstrual policies. This represents a significant challenge, given that it is deeply rooted in hegemonic constructions of masculinity, gender norms and relations. The commitment of men and PNM towards menstrual equity has the potential to drive socio-political changes that are necessary to address menstrual inequities. Finally, interdisciplinary gender and intersectional research should continue to investigate how men and PNM could contribute to menstrual (in)equity and health.

## Supporting information

S1 File Interview topic guide. (DOCX)

S2 File Critical Appraisal Skills Programme (CASP) criteria. (DOCX)

## Abbreviations

PNM: People who do not menstruate
PWM: People who menstruate
