# Supplementary material for: “If everyone comes together, many things can be changed”: A qualitative study on men’s perspectives on menstrual health and equity in the Barcelona area (Spain)
Source: PLoS One. 2025 Feb 27;20(2):e0312685. doi: 10.1371/journal.pone.0312685 (PMC11867308; doi:10.1371/journal.pone.0312685)
Supplement: S1 File — (DOCX) [file pone.0312685.s001.docx]

**S1 Table. Interview topic guide.**

| **Objectives** | **Questions** |
| --- | --- |
| Introduction | 1. How do you describe yourself? |
| Explore the sources of learning and types of information | 1. Do you remember the first time you heard about menstruation? 2. Where do you think you learned about the menstrual cycle and menstruation?   ● What do you think about menstrual education at school? (age/topics covered)  ● What influence do you think the media has had on what you have learned?  ● Do you think your information about menstruation has changed over time?   1. What do you think about the adequacy of the information you have received about menstruation and the menstrual cycle?   ● Are there any topics you would like to explore in more depth?   1. Do you think that information about menstruation and the menstrual cycle can be useful for men and PNM? |
| Explore the conceptualization and attitudes towards menstruation | 1. Do you think that there are positive aspects to the menstrual cycle and menstruation? And negative? 2. During the menstrual cycle, women/PWM may experience changes on a physical and emotional level. Which changes do you think are the most common?   ● What do you think about these changes?  ● Do you think that women could exaggerate these experiences?  ● How do you think these changes affect men/PNM?   1. When someone around you is menstruating (ex. mother, friend, partner, daughter), do they ask you for help?   ● If she/they does not, do you care more for her/them?  ● What do you do to take care of her/them?   1. Do you think menstruation can be a disadvantage for women/PWM compared to men/PNM? |
| Explore the taboo, stigma and discrimination associated with menstruation | [Show photograph 1, marathon runners]   1. Could you describe this photograph? 2. What do you feel looking at it? 3. Some men/PNM tell us that they are disgusted by menstrual blood. Can you tell us if you have had any similar experiences?   ● If you have had sex with women/PWM, are you uncomfortable having sex during menstruation? Do you try to avoid the bloodiest days?  ● Do you talk about menstruation with women/PWM? And with other men/PNM?  ● Do you consider menstruation to be a taboo topic? Why do you think menstruation is a taboo subject?   1. Some men/PNM have told us about situations in which a woman/PWM has been ridiculed in public (e.g. at school or at work). Have you ever witnessed someone being ridiculed because of their menstruation?  - How have you acted when someone has been ridiculed? |
| Identifying opportunities to engage men and people who do not menstruate in improving menstrual health and reducing inequities in menstruation | [Show photograph 2, Mexican congresswomen]   1. Could you describe this photograph? 2. What do you feel looking at it? 3. What do you think about measures to reduce the price of menstrual products?   ● Do you think that they should be provided free of charge/subsidized to certain groups?  ● Have you ever met women/PWM who have problems buying menstrual products?   1. In addition to these policies, other measures could also be applied to improve menstrual health. Can you think of any measures that might be needed? 2. What do you think about policies that could be applied to the menstrual work environment? (e.g. flexible working hours, teleworking, menstrual leave...). 3. Do you think that policies to strengthen menstrual education would be necessary? 4. Do you consider other measures? 5. What could be the role of men/PNM in menstrual (social and political) changes? |
