# Supplementary material for: “If everyone comes together, many things can be changed”: A qualitative study on men’s perspectives on menstrual health and equity in the Barcelona area (Spain)
Source: PLoS One. 2025 Feb 27;20(2):e0312685. doi: 10.1371/journal.pone.0312685 (PMC11867308; doi:10.1371/journal.pone.0312685)
Supplement: S2 File — (DOCX) [file pone.0312685.s002.docx]

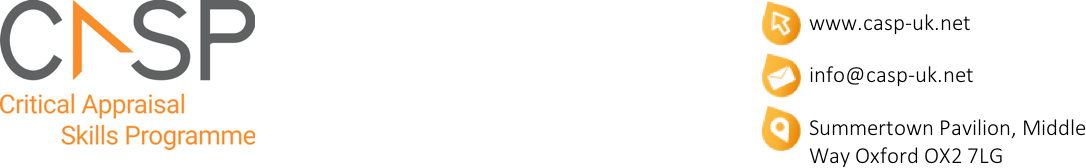


# CASP Checklist: 10 questions to help you make sense of a Qualitative research

How to use this appraisal tool: Three broad issues need to be considered when appraising a qualitative study:

# Are the results of the study valid? (Section A) What are the results? (Section B) Will the results help locally? (Section C)


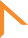

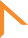

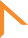


The 10 questions on the following pages are designed to help you think about these issues systematically. The first two questions are screening questions and can be answered quickly. If the answer to both is “yes”, it is worth proceeding with the remaining questions. There is some degree of overlap between the questions, you are asked to record a “yes”, “no” or

# “can’t tell” to most of the questions. A number of italicised prompts are given after each question. These are designed to remind you why the question is important. Record your reasons for your answers in the spaces provided.

About: These checklists were designed to be used as educational pedagogic tools, as part of a workshop setting, therefore we do not suggest a scoring system. The core CASP checklists (randomised controlled trial & systematic review) were based on JAMA 'Users’ guides to the medical literature 1994 (adapted from Guyatt GH, Sackett DL, and Cook DJ), and piloted with health care practitioners.

# For each new checklist, a group of experts were assembled to develop and pilot the checklist and the workshop format with which it would be used. Over the years overall adjustments have been made to the format, but a recent survey of checklist users reiterated that the basic format continues to be useful and appropriate.

Referencing: we recommend using the Harvard style citation, i.e.: *Critical Appraisal Skills Programme (2018). CASP (insert name of checklist i.e. Qualitative) Checklist. [online] Available at: URL. Accessed: Date Accessed.*

©CASP this work is licensed under the Creative Commons Attribution – Non-Commercial- Share A like. To view a copy of this license, visit <http://creativecommons.org/licenses/by-nc-> sa/3.0/ [www.casp-uk.net](http://www.casp-uk.net/)

Critical Appraisal Skills Programme (CASP) part of Oxford Centre for Triple Value Healthcare Ltd [www.casp-uk.net](http://www.casp-uk.net/)

**Paper for appraisal and reference:** García -Egea A, Jacques-Aviñó C, Berenguera A, Holst AS, Pinzón-Sanabria D, Munrós-Feliu J, et al. Conceptualization and perspectives of men in equity and menstrual health in the Barcelona area: a qualitative and exploratory study. In prep.

Section A: Are the results valid?

1. Was there a clear statement of the aims of the research?

Comments: The study aimed at exploring sociocultural meanings and attitudes towards menstruation in men and people who do not menstruate between 18-55 years old in Barcelona and surrounding areas. Finally, the study only involved people identified as men. The study was addressed because it is an understudied issue globally and in our geographical context.

1. Is a qualitative methodology appropriate?

Yes

Can’t Tell

No

Yes

Can’t Tell

No

HINT: Consider

- what was the goal of the research

| X |
| --- |
|  |
|  |

- - why it was thought important
    - its relevance

HINT: Consider

| X |
| --- |
|  |
|  |

- - If the research seeks to interpret or illuminate the actions and/or subjective experiences of research participants
    - Is qualitative research the right methodology for addressing the

research goal

Comments: The qualitative methodology allows us to explore narratives and discourses in a more holistic way, allowing participants to explain their perceptions of the world and how they construct it. Therefore, this type of research is suitable for exploring the purpose of the study.

Is it worth continuing?

1. Was the research design appropriate to address the aims of the research?

Yes

Can’t Tell

No

HINT: Consider

- if the researcher has justified the research design (e.g. have they discussed how they decided which

| X |
| --- |
|  |
|  |

method to use)

Comments: The design and theoretical approach taken to conduct the research have been specified and justified in the article.

1. Was the recruitment strategy appropriate to the aims of the research?

Yes

Can’t Tell

No

HINT: Consider

- If the researcher has explained how the

| X |
| --- |
|  |
|  |

participants were selected

- - If they explained why the participants they selected were the most appropriate to provide access to the type of knowledge sought by the study
    - If there are any discussions around recruitment (e.g. why some people

chose not to take part)

Comments: Due to COVID-19 pandemic, the recruitment strategy could not be followed and we expected. It was possible to recruit participants with different socio-demographic characteristics to generate discourse diversity. However, there could have been done more to include non-binary, intersexual and trans people.

1. Was the data collected in a way that addressed the research issue?

Yes

Can’t Tell

No

HINT: Consider

- If the setting for the data collection was

| X |
| --- |
|  |
|  |

justified

- If it is clear how data were collected (e.g. focus group, semi-structured interview

etc.)

- If the researcher has justified the methods

chosen

- - If the researcher has made the methods explicit (e.g. for interview method, is there an indication of how interviews are conducted, or did they use a topic guide)
    - If methods were modified during the study. If so, has the researcher explained how and why
      - If the form of data is clear (e.g. tape recordings, video material, notes etc.)
        - If the researcher has discussed

saturation of data

Comments: Semi-structured individual interviews were conducted because they are more appropriate than group discussions to address taboo and stigmatized topics. In addition, photo-elicitation techniques facilitate conversation on complex topics and give rise to answers that don’t emerge trough verbal or written communication.

Data was collected through audio recordings, with the prior consent of the participants, so that interviewees’ speeches could be transcribed.

1. Has the relationship between researcher and participants been adequately considered?

Yes

Can’t Tell

No

HINT: Consider

- - If the researcher critically examined their own role, potential bias and influence during (a) formulation of the research questions (b) data collection, including sample recruitment and choice of

| X |
| --- |
|  |
|  |

location

- How the researcher responded to events during the study and whether they considered the implications of any changes in the

research design

Comments: Authors critically examined their role and influence during data collection and subsequent analysis. This issue has been taken into account during the development of the study in order to minimize the associated biases.

Section B: What are the results?

1. Have ethical issues been taken into consideration?

Yes

Can’t Tell

No

HINT: Consider

- If there are sufficient details of how the research was explained to participants for the reader to assess whether ethical standards were maintained

| X |
| --- |
|  |
|  |

- - If the researcher has discussed issues raised by the study (e.g. issues around informed consent or confidentiality or how they have handled the effects of the study on the participants during and after the

study)

- - - If approval has been sought from

the ethics committee

Comments: The study obtained the necessary ethical approval on 20th November 2019 (Ref 19/178-P). The administration of informed consent to all participants, both verbal and written, was provided prior to the start of the interviews. All participants were informed of anonymity, confidentiality, and the right to retain information, as well as to withdraw from the study or delete their data prior to analysis.

1. Was the data analysis sufficiently rigorous?

Yes

Can’t Tell

No

HINT: Consider

- - If there is an in-depth description of the

| X |
| --- |
|  |
|  |

analysis process

- If thematic analysis is used. If so, is it clear how the categories/themes were derived

from the data

- Whether the researcher explains how the data presented were selected from the original sample to demonstrate the analysis

process

- If sufficient data are presented to support

the findings

- - To what extent contradictory data are

taken into account

- Whether the researcher critically examined their own role, potential bias and influence during analysis and selection of data for

presentation

Comments: Data was analysed through a thematic analysis, following the different steps that characterize this type of analysis. The initial coding has been triangulated with two researchers of the team. Also, the categories and subcategories generated have been discussed with much of the research team, in order to reach to the final analysis framework.

1. Is there a clear statement of findings?

Yes

Can’t Tell

No

HINT: Consider whether

- - If the findings are explicit

| X |
| --- |
|  |
|  |

- If there is adequate discussion of the evidence both for and against the researcher’s arguments
  - If the researcher has discussed the credibility of their findings (e.g. triangulation, respondent validation, more

than one analyst)

- If the findings are discussed in relation to

the original research question

Comments: The article specifies and discuss the results found. Also, the conclusions reached through the research and practice recommendation are explained.

| Section C: Will the results help locally? |  |
| --- | --- |
| 10. How valuable is the research? | HINT: Consider   - If the researcher discusses the contribution the study makes to existing knowledge or understanding (e.g. do they consider the findings in relation to current practice or policy, or relevant research-   based literature   - If they identify new areas where research   is necessary   - If the researchers have discussed whether or how the findings can be transferred to other populations or considered other ways the research may be used |
| Comments: This is a relevant research as menstrual health and (in) equity remain under-studied issues. The literature mostly encompasses the experience of women and people who menstruate, but there is a lack of attention to the perspectives that men and people who do not menstruate have about this phenomenon. Therefore, this study has focused on the exploration of men’s discourses. | |
